# The Correlation Between Vitamin D Deficiency and Anemia: A Systematic Review

**DOI:** 10.7759/cureus.89428

**Published:** 2025-08-05

**Authors:** Mohammed Alpakra, Nazim F Hamed, Ammar Alfaki, Danya Mohammed Zuhair AlKabbani

**Affiliations:** 1 Oncology and Hematology Department, Armed Forces Hospital Southern Region, Khamis Mushait, SAU; 2 General Pediatrics, Security Forces Hospital, Dammam, SAU; 3 Internal Medicine, Security Forces Hospital, Dammam, SAU; 4 Adult Hematologist, Security Forces Hospital, Dammam, SAU

**Keywords:** 25-hydroxyvitamin d, anemia, iron deficiency anemia, vitamin d deficiency, vit d

## Abstract

Vitamin D deficiency (VDD) and anemia are common public health problems around the world. Recent data points to a biological connection between these disorders, especially in relation to vitamin D's function in controlling iron and hepcidin metabolism. The study aims to do a comprehensive review of the literature on the relationship between adult populations' anemia and VDD. PubMed, Web of Science, Scopus, and Embase were all thoroughly searched. Included were observational studies evaluating the connection between adult anemia and vitamin D levels. Six studies were included, with a total of 10,953 participants, and 2690 (24.6%) of them were men. The range of VDD prevalence was 18.8% to 87.1%, with a total prevalence of 5479 (50%). Among the participants with VDD, 2041 (37.2%) had anemia. Anemia was shown to be more common in those with VDD in every study. Mean hemoglobin, ferritin, and red blood cell counts were consistently lower in VDD groups. In the majority of studies, the connection persisted even after controlling for covariates. There is strong evidence supporting an association between VDD and increased risk of anemia. These results support more interventional research to evaluate the potential benefits of including vitamin D screening in anemia workups and the benefits of vitamin D supplementation in anemic persons.

## Introduction and background

Both VDD and anemia are widespread across the globe [1,2]. Because of their frequent co-occurrence in different groups, the pathogenesis of anemia has been thought to be influenced by insufficient vitamin D [3]. One widely accepted theory is that vitamin D plays a crucial role in regulating hepcidin, the principal hormone governing systemic iron regulation [4].

Vitamin D is available from limited dietary sources, including naturally occurring options like oily fish, eggs, and mushrooms, in addition to fortified foods like vitamin pills and milk [5]. Nevertheless, the majority of individuals rely on the body’s own synthesis of vitamin D. This happens when sunlight's ultraviolet (UV) energy changes. Vitamin D is produced by the skin from 7-dehydrocholesterol [6]. Several factors can hinder this UV-driven synthesis, such as seasonal variation, geographic latitude, time of day, skin pigmentation, and the extent of skin covered due to cultural or religious practices. After entering the body, no matter where it comes from, the main form that circulates and the most precise method of measuring vitamin D levels is 25-hydroxyvitamin D (25OHD), which is created when vitamin D is hydroxylated in the liver [7,8]. However, there is ongoing debate over the optimal threshold for sufficiency (ranging from 20 to 100 nmol/L), contributing to inconsistent prevalence rates of deficiency.

VDD and anemia are two globally prevalent conditions that significantly impact public health. Recent data points to a potential physiological connection between the two, especially given vitamin D's function in controlling the hormone hepcidin, which is essential for iron metabolism. VDD and decreased hemoglobin levels have been found to co-occur in a number of observational studies, indicating a potential causative association. However, findings across studies remain inconsistent, and the underlying mechanisms are not yet fully clarified. A systematic review is needed to consolidate existing data, evaluate the strength of the association, and explore the biological plausibility of this correlation. Understanding this relationship could inform preventive strategies and integrated treatment approaches, particularly in vulnerable populations.

In order to ascertain whether a significant correlation exists, identify potential underlying mechanisms, and highlight gaps in the literature that require additional research, this review attempts to methodically examine and synthesize the available data on the relationship between VDD and anemia.

## Review

Methods

To maintain scientific integrity and openness, the Preferred Reporting Items for Systematic Reviews and Meta-Analyses (PRISMA) guidelines were followed in the conduct of this systematic review [9]. The objective was to examine the correlation between VDD and anemia in adult populations through a comprehensive examination of observational research.

Search strategy

Table 1 shows the search strategy.

**Table 1:** Elements of the search strategy in a structured format

Component	Details
Databases	PubMed, Web of Science, Scopus, Embase
Time Frame	From inception to the most recent update
Language	English
Population	Human studies
Search Terms	Vitamin D: "Vitamin D," "cholecalciferol," "25-hydroxyvitamin D," "vitamin D deficiency" Anemia: "Anemia," "hemoglobin," "iron deficiency" Association: "Association," "Correlation"
Boolean Operators	AND, OR (e.g., ("Vitamin D" OR "cholecalciferol") AND ("Anemia" OR "hemoglobin") AND ("Association" OR "Correlation")
Additional Search	Manual screening of reference lists from included studies

Study selection and eligibility criteria

After screening all titles and abstracts, two impartial reviewers went on to examine the complete texts of any papers that could be suitable. A third reviewer was consulted, or a consensus was reached to settle disagreements.

The inclusion criteria for this review encompassed studies that investigate the relationship between vitamin D levels and anemia in individuals aged 18 and older, utilizing cross-sectional, cohort, and case-control observational designs. Eligible studies were required to report quantitative data on vitamin D status and anemia-related outcomes, such as hemoglobin levels and anemia prevalence, and must have been published within the last 10 years (2015-2025) in English-language peer-reviewed journals. Conversely, the exclusion criteria ruled out research focused on children, pregnant women, or specific populations with underlying chronic conditions (e.g., cancer, chronic kidney disease, sickle cell disease). Additionally excluded were reviews, editorials, conference abstracts, and case reports, as well as non-human or in vitro studies and studies that did not provide extractable data on both vitamin D and anemia outcomes.

Data extraction

To streamline the screening and selection process, Rayyan Systems Inc., Cambridge, MA, US, from Qatar Computing Research Institute (QCRI) [10] was employed. A standardized data extraction form was used to gather essential information, including the study's features, such as the authors, year of publication, country, and study design, as well as details on sample size and demographics. Additionally, the form captured definitions and measurement methods for vitamin D status and anemia and reported associations like correlation coefficients, odds ratios, and mean differences, along with adjustments for confounding variables and relevant statistical outcomes.

Bias assessment risk

The ROBINS-I tool for interventions [11] was used to evaluate the methodological quality and possible sources of bias in all qualifying non-randomized trials. Randomized controlled trials were conducted using the Cochrane Risk of Bias Tool when applicable (Table 2). 

**Table 2:** Risk of bias assessment using ROBINS-I

Study ID	Bias due to confounding	Bias in the selection of participants into	Bias in the classification of interventions	Bias due to deviations from the intended interval	Bias due to missing data	Bias in the measurement of outcomes	Bias in the selection of reported result	Overall bias
Smith et al., 2015 [12]	Low	Low	Low	Low	Low	Low	Mod	Low
Nur-Eke & Özen, 2020 [13]	Low	Low	Mod	Low	Low	Low	Mod	Low
Zouine et al., 2024 [14]	Mod	Mod	Mod	Low	Low	Low	Mod	Moderate
Alaasswad et al., 2024 [15]	Mod	Loe	Low	Low	Low	Mod	Mod	Moderate
Alam et al., 2023 [16]	Mod	Low	Low	Low	Mod	Mod	Mod	Moderate
Bhushan et al., 2024 [17]	Crit	Mod	Crit	Low	Low	Mod	Low	Critical

Results

**Figure 1:**
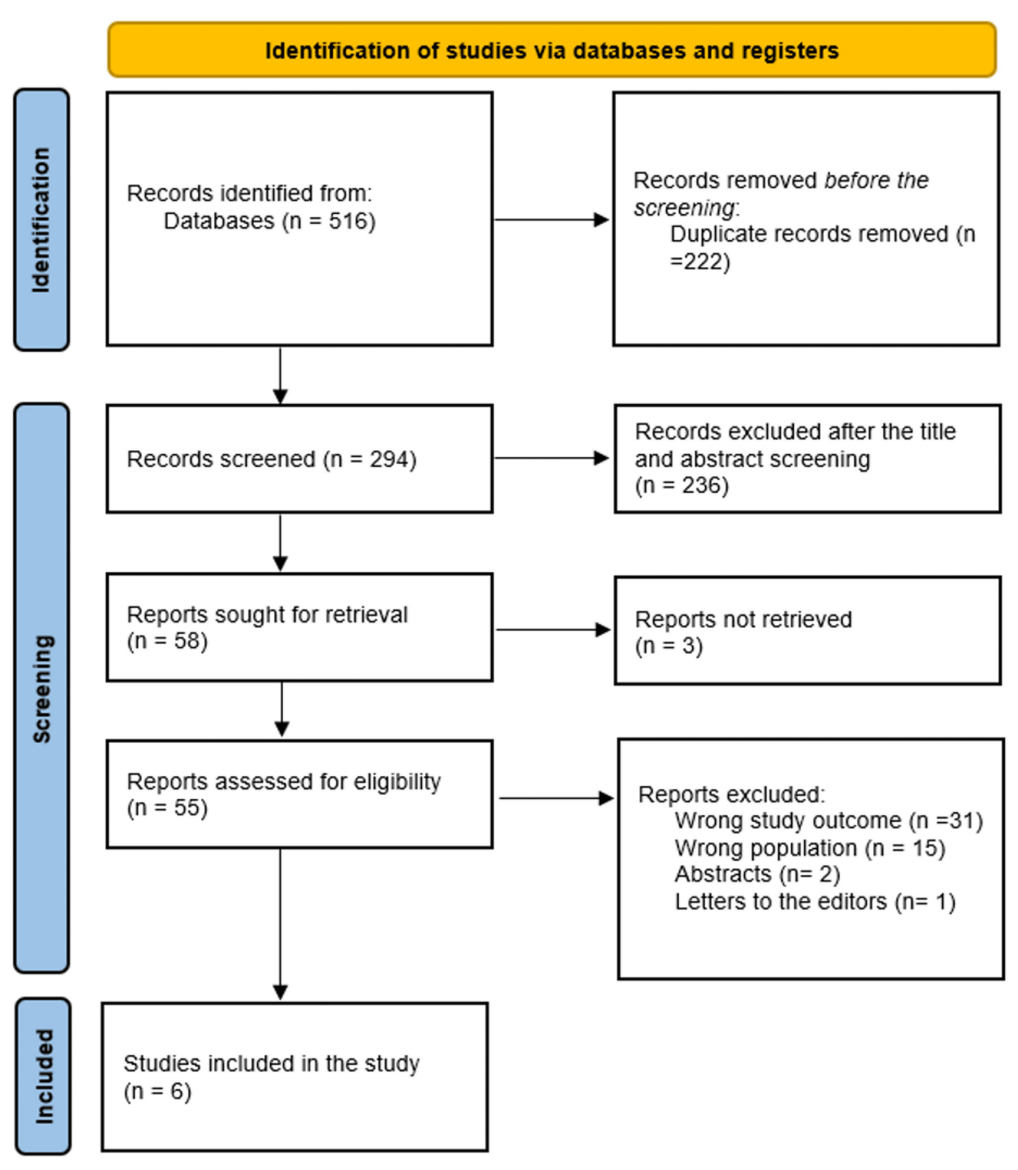
The search summary is illustrated in the PRISMA flowchart The image is created by the author.

The search process initially identified 516 publications (Figure 1). A total of 294 studies were selected for screening based on their abstracts and titles after 222 duplicates were eliminated. Of these, 55 full-text publications were left for a comprehensive evaluation. After 236 did not meet the qualifying requirements. Six papers were ultimately chosen for evidence synthesis and analysis after meeting the inclusion criteria.

Clinical results and sociodemographic 

Of the 10,953 individuals in the six included trials, 2690 (24.6%) were men. Every study that was included was cross-sectional [12-17]. Two studies were conducted in India [16,17], one in the USA [12], one in Turkey [13], one in Morocco [14], and one in Libya [15] (Table 3).

**Table 3:** Summary of sociodemographics from the included studies.

Study ID	Country	Study design	Sample size	Mean age (SD)	Males (%)
Smith et al., 2015 [12]	USA	Cross-sectional	638	48.3 (10.9)	206 (32.3%)
Nur-Eke & Özen, 2020 [13]	Turkey	Cross-sectional	9,590	43.13 (13)	2,395 (25%)
Zouine et al., 2024 [14]	Morocco	Cross-sectional	463	18-49 (range)	0
Alaasswad et al., 2024 [15]	Libya	Cross-sectional	62	20.6 (3.2)	35 (56.5%)
Alam et al., 2023 [16]	India	Cross-sectional	100	47.7 (9.1)	28 (28%)
Bhushan et al., 2024 [17]	India	Cross-sectional	100	45.5 (8.06)	26 (26%)

Clinical outcomes 

The prevalence of VDD ranged from 18.8% [12] to 87.1% [15], with a total prevalence of 5479 (50%). Among the participants with VDD, 2041 (37.2%) had anemia.

The primary conclusions drawn from the research consistently point to a strong link between VDD and a higher risk of iron deficiency anemia (IDA). One study demonstrated that those with low vitamin D levels had a considerably higher frequency of anemia, even after controlling for confounding variables. This risk was particularly high in those with anemia linked to inflammation, which may indicate that vitamin D plays a part in immune-modulated anemia pathways [12].

Another study emphasized that individuals with VDD exhibited substantially lower serum levels of hemoglobin, iron, and ferritin compared to those with sufficient vitamin D. Additionally, it was shown that those with anemia had steadily declining mean vitamin D concentrations, iron deficiency, and IDA, confirming a gradient association between hematological health and vitamin D levels [13].

Further investigation showed that VDD was substantially linked to decreased levels of ferritin, hemoglobin, hematocrit, and red blood cell count, indicating that vitamin D may be necessary for both erythropoiesis and iron storage [14]. Similarly, a study on young adults indicated that VDD was prevalent and closely associated with higher rates of anemia, reinforcing the potential public health importance of vitamin D screening in this age group [15].

Another dataset showed that nearly half of the individuals with VDD had anemia, and the authors concluded that this vitamin deficiency is significantly correlated with anemia risk [16]. Finally, an additional study reported a significantly lower mean hemoglobin level in the VDD group (9.62 g/dL) versus the control group (10.66 g/dL), verifying the connection between decreased hematologic markers and low vitamin D levels [17]. Table 4 shows the clinical outcomes.

**Table 4:** Summary of clinical outcomes from the included studies.

Study ID	Anemia definition (g/dL)	VDD definition (ng/mL)	Type of anemia	VDD (%)	Prevalence of anemia in VDD	Main
Smith et al., 2015 [12]	13 g/dL for males and, 12 g/dL for females	<20	IDA	116 (18.8%)	18 (15.5%)	Specifically, Black people with low vitamin D levels were much more likely to develop anemia than those with adequate vitamin D, even after adjusting for other relevant variables. White people did not exhibit this relationship. Additionally, the risk was particularly high for inflammation-related anemia.
Nur-Eke & Özen, 2020 [13]	< 13 g/dL in males and < 12 g/dL in non-pregnant females	< 20	IDA	4,865 (50.7%)	1.836 (56.8%)	Serum ferritin, iron, and hemoglobin levels were considerably lower in those with low vitamin D levels than in those with adequate vitamin D. Furthermore, those with anemia (17.4 ng/mL), iron deficiency (18.2 ng/mL), and IDA (16.6 ng/mL) had significantly lower mean vitamin D levels than those without these diseases.
Zouine et al., 2024 [14]	<12	< 20	IDA	334 (72.1%)	101 (93.5%)	VDD was strongly linked to reduced levels of hemoglobin, hematocrit, red blood cells, and ferritin, suggesting a key role of vitamin D in red blood cell production and iron storage.
Alaasswad et al., 2024 [15]	<12	<20	IDA	54 (87.1%)	26 (48.1%)	Young individuals frequently suffer from VDD, which is strongly linked to a greater incidence of anemia.
Alam et al., 2023 [16]	<12	<30	IDA	60 (60%)	29 (48.3%)	The results point to a direct link between VDD and a higher incidence of anemia.
Bhushan et al., 2024 [17]	<13	<30	IDA	50 (50%)	31 (62%)	The average hemoglobin level was notably lower in the VDD group (9.62 g/dL) compared to the control group (10.66 g/dL). These findings support the conclusion that VDD is linked to an increased risk of anemia.

Discussion

This systematic review highlights a consistent and significant correlation between VDD and IDA in adult populations. Across the included studies, individuals with low vitamin D levels were found to have reduced hemoglobin concentrations and higher rates of anemia. This association was evident even after controlling for potential confounders such as inflammation, race, and dietary differences, suggesting a possible biological link beyond coincidental comorbidity. According to the findings, vitamin D could be involved in regulating hepcidin, a hormone that is essential for iron metabolism, and the effects it has on erythropoiesis and iron availability.

When Lima et al. initially employed the search approach, they discovered 985 studies in total [18]. After screening and applying the inclusion criteria, 17 papers, 11 cohort studies, three case-control studies, and three cross-sectional studies, were deemed suitable for the systematic review. Eight of these investigations, totaling 6,530 pregnant women, were included in the meta-analysis. Anemia and vitamin D insufficiency were shown to be statistically significantly correlated, with pregnant women who were vitamin D deficient having a 61% increased risk of anemia (OR = 1.61; 95% CI: 1.41-1.83; I² = 48%). According to these results, VDD may increase the risk of anemia during pregnancy [18].

Interestingly, research in this study revealed that those with iron deficiency and anemia had significantly lower mean blood vitamin D levels, further supporting the hypothesis of a dose-response relationship. Several reports also indicated lower ferritin, red blood cell counts, and hematocrit levels in VDD groups, providing mechanistic plausibility for the observed associations. These consistent patterns across diverse populations reinforce the importance of investigating vitamin D not only as a skeletal health nutrient but also as a modulator of hematologic function. 

Liu et al. showed a substantial correlation (odds ratio of 2.25, 95% CI: 1.47-3.44) between VDD and an elevated incidence of anemia. The included studies did, however, exhibit significant heterogeneity (I² = 84%, p < 0.001). Subgroup and sensitivity analyses supported the findings' robustness in spite of this heterogeneity, and no evidence of publication bias was found. These findings imply that an increased incidence of anemia is linked to VDD. However, the existence of heterogeneity emphasizes the necessity of further well-planned research to fully comprehend the nature and processes of this association [19].

Vitamin D is believed to influence hemoglobin levels by supporting red blood cell formation. It has been demonstrated that the active form of 1,25-dihydroxyvitamin D (1,25OH₂D) increases erythropoiesis by promoting the growth of erythroid progenitor cells and intensifying the effects of erythropoietin [20,21]. Increased parathyroid hormone (PTH) levels in renal illness can have a detrimental effect on erythropoiesis and result in lower hemoglobin levels. Vitamin D may counteract this by inhibiting PTH secretion, thereby supporting red blood cell production [22].

Nonetheless, the most widely supported mechanism by which vitamin D may guard against anemia is its ability to modulate inflammation. According to certain theories, vitamin D has a critical role in regulating the hormone hepcidin, which is necessary for systemic iron metabolism [23]. Chronic inflammatory conditions, whether brought on by infections, autoimmune illnesses, cancer, obesity, or behaviors like smoking and drinking, can decrease hemoglobin synthesis by decreasing the bioavailability of iron [24].

Vitamin D may attenuate this process by lowering pro-inflammatory cytokines and suppressing hepcidin levels, which are crucial for iron homeostasis. Inflammatory cytokines, especially interleukin-6, impair erythropoiesis, reduce erythropoietin efficacy, and shorten red blood cell survival. IL-6 also activates the hepcidin gene via the JAK2/STAT3 pathway, promoting hepcidin synthesis in the liver [25]. After being created, hepcidin attaches itself to enterocytes, macrophages, and ferroportin, the sole known cellular iron exporter found in hepatocytes, to restrict the quantity of iron available for the production of red blood cells and to stop iron from being discharged into the bloodstream [26].

Vitamin D can inhibit the transcription of hepcidin, particularly when it is in its active state (1,25OH₂D). It has the ability to attach to the hepcidin gene's promoter region's vitamin D response element (VDRE). This reduction in hepcidin allows ferroportin to continue exporting iron into circulation, making it available for erythropoiesis [26].

Despite the growing body of evidence, it remains difficult to determine whether VDD is a causal factor in anemia or simply a coexisting condition driven by shared socioeconomic, dietary, or environmental risk factors. Most of the studies included were observational in nature, and while they provide compelling associations, they cannot establish causality. Furthermore, the use of varying cut-off values for both VDD and anemia, as well as differences in the populations studied, may introduce heterogeneity in the results.

Strengths

This review's strict adherence to PRISMA principles and thorough search across several databases, which reduced the possibility of overlooking pertinent research, are two of its main strengths. The inclusion of only adult populations and the exclusion of individuals with chronic illnesses such as chronic kidney disease (CKD), sickle cell disease (SCD), or pregnancy allowed for a more comprehensive examination of the relationship between vitamin D and anemia in the general adult population. This helped to minimize potential confounding. Furthermore, the extraction and synthesis of quantitative data enhanced the interpretability of the findings.

Limitations

Nonetheless, this review is subject to several limitations. The majority of included studies were observational, limiting causal inference. The heterogeneity in the definitions of VDD and anemia across studies may also affect comparability. Additionally, certain studies failed to account for all possible confounding factors, which might affect both vitamin D levels and hematologic results. These factors include food consumption, inflammatory indicators, and socioeconomic status. Lastly, publication bias cannot be excluded, as studies reporting positive associations may be more likely to be published than those with null findings.

## Conclusions

This systematic review provides compelling evidence of a consistent association between VDD and increased risk of IDA in adults. The findings underline the need for more research on vitamin D's role in hematologic health and encourage the inclusion of vitamin D testing in the diagnostic workup of anemia. To elucidate the underlying association and ascertain if vitamin D supplementation might be a useful tactic in the prevention or treatment of anemia, more longitudinal and interventional research is necessary.
